# Molecular Profiles of Cell-to-Cell Variation in the Regenerative Potential of Mesenchymal Stromal Cells

**DOI:** 10.1155/2019/5924878

**Published:** 2019-09-17

**Authors:** Kim C. O'Connor

**Affiliations:** ^1^Department of Chemical and Biomolecular Engineering, Tulane University, New Orleans, Louisiana, USA; ^2^Center for Stem Cell Research and Regenerative Medicine, Tulane University School of Medicine, New Orleans, Louisiana, USA

## Abstract

Cell-to-cell variation in the regenerative potential of mesenchymal stromal cells (MSCs) impedes the translation of MSC therapies into clinical practice. Cellular heterogeneity is ubiquitous across MSC cultures from different species and tissues. This review highlights advances to elucidate molecular profiles that identify cell subsets with specific regenerative properties in heterogeneous MSC cultures. Cell surface markers and global signatures are presented for proliferation and differentiation potential, as well as immunomodulation and trophic properties. Key knowledge gaps are discussed as potential areas of future research. Molecular profiles of MSC heterogeneity have the potential to enable unprecedented control over the regenerative potential of MSC therapies through the discovery of new molecular targets and as quality attributes to develop robust and reproducible biomanufacturing processes. These advances would have a positive impact on the nascent field of MSC therapeutics by accelerating the development of therapies with more consistent and effective treatment outcomes.

## 1. Introduction

Mesenchymal stromal cells (MSCs) are the most common stem cell therapy in clinical trials [1]. This popularity traces back to the groundbreaking research of Friedenstein et al. who identified colony-forming unit fibroblasts (now known as MSCs) in bone marrow [2]. This early research demonstrated that MSCs have a remarkable capacity to regenerate osseous tissue *in vivo* [3]. MSCs have been given several names over the years, including marrow stromal cells and multipotent stromal cells [4, 5], and have been isolated from many tissues, such as adipose and the umbilical cord [6, 7]. The current popularity of MSCs as a stem cell therapy reflects their broad regenerative properties to home to the site of injury [8], undergo extensive proliferation [9], exhibit multipotency [10], regulate the immune system [11], and secrete trophic factors [12]. The therapeutic applications of these pleiotropic cells are vast. Clinical trials with MSCs are underway to treat skeletal defects, graft-vs.-host disease, and cardiovascular disorders, to name a few [13].

A barrier to realize the therapeutic potential of MSCs is their intrinsic heterogeneity. MSCs are a composite of cell progenitors at different states of lineage commitment [14, 15] and cellular aging [16, 17]. Cellular heterogeneity is ubiquitous across MSC cultures harvested from different species and tissues [18–20]. Cell-to-cell variation in MSC function initiates *in vivo* in the stem cell niche [21], is evident within single-cell-derived MSC colonies [22], and is exacerbated by replicative stress during *ex vivo* cultivation [16]. Cell subsets within heterogeneous MSC cultures vary in their regenerative potential, including proliferation potential [23, 24] and potency [10, 14]. Cellular heterogeneity has impacted the effectiveness of MSC therapies in animal models to repair bone, cartilage, and the heart, among other tissues [25–27]. This heterogeneity has been cited as a possible factor contributing to the variability in treatment outcomes of MSC therapies in clinical trials [13, 28, 29]. Variation in the regenerative potential among cell subsets in MSC cultures may confound trial results and slow, if not arrest, the translation of an MSC therapy into clinical practice.

There is a critical need for molecular profiles of MSC heterogeneity to manufacture effective MSC therapies. This review highlights advances to elucidate cell surface markers and global signatures that identify cell subsets with specific regenerative properties in heterogeneous MSC cultures. Molecular profiles of MSC heterogeneity will enable cell enrichment and quality control assessment during the manufacturing of MSC therapies to standardize cell composition. In addition, they will help identify new molecular targets to regulate the regenerative potential of MSCs. Molecular profiles of MSC heterogeneity are expected to make a positive impact on the nascent field of MSC therapeutics by accelerating the development of therapies with more consistent and effective treatment outcomes.

## 2. Proliferation Potential

MSCs are a rare population of progenitors in adult tissue [10] and are expanded *ex vivo* to obtain a sufficient amount of cells for clinical applications [30]. Cell-to-cell variation in the proliferation potential of MSCs gives rise to cell population dynamics during *ex vivo* expansion that alters the composition of cell subsets in culture and, in turn, may impact the efficacy of MSC therapies [31]. Heterogeneity in the proliferation potential of MSC cultures was first reported in morphologically distinct subsets of small, rapidly dividing cells and large, slowly dividing cells [23, 24]. We and others have validated this functional heterogeneity in proliferation potential with single-cell-derived colonies that originated from a common, parental MSC culture [15, 32, 33].

### 2.1. Cell Surface Markers of Proliferation Potential

A focus of ongoing research on MSC heterogeneity is to elucidate an immunophenotype of proliferation potential. Cell surface markers enable noninvasive and nondestructive isolation of specific cell subsets from MSC cultures for research and clinical applications. The International Society for Cellular Therapy has specified that human MSCs must express CD73, CD90, and CD105 [34]. We and others observed little to no variation in surface expression of these biomarkers between rapidly and slowly dividing cells in cultures of human bone marrow-derived MSCs (hBM-MSCs) [17, 32, 35]. The inability of the standard MSC immunophenotype to detect specific cell subsets in MSC cultures demonstrates the need for new surface markers of MSC heterogeneity.

Several cell surface markers have been identified to isolate MSCs with high colony-forming efficiency from tissues (Table 1). Of these, the 75 kDa endothelial antigen STRO-1 [36, 37], heterotypic intercellular adhesion molecule CD146 [38], and the low-affinity nerve growth factor receptor CD271 [39] are among the most investigated isolation markers. STRO-1 was named for its ability to isolate the stromal fraction of human bone marrow [37]. CD146 is a pericyte marker [40], and its expression by MSCs is consistent with a perivascular origin for the postnatal MSC niche [41]. CD271^+^ stroma emerges in fetal bone marrow before the onset of hematopoietic activity [42], suggesting that CD271 detects primitive mesenchymal cells. Positive selection with any of these surface markers recovers most, if not all, of the colony-forming unit fibroblasts present in human bone marrow aspirates [37–39]. MSC surface markers exhibit tissue-specific expression. Consider CD271, which is an effective marker to isolate hBM-MSCs and human adipose-derived (hAD) MSCs [39, 43]; however, it is weakly expressed on MSCs from human Wharton's jelly (hWJ) [44]. Once isolated, primary MSC cultures are functionally heterogeneous. As an example, Simmons and Torok-Storb reported that colony-forming unit fibroblasts accounted for only 1% of the STRO-l^+^/glycophorin A^−^ fraction of human bone marrow [37]. When MSCs are expanded *ex vivo*, some isolation markers are rapidly downregulated [45], as is the case for CD271 [46].

Surface markers that detect cellular heterogeneity in cultured MSCs enable quality control of MSC therapies during *ex vivo* expansion. One category of markers for cultured MSCs is a subset of isolation markers, including CD146 and stage-specific embryonic antigen-4 (SSEA-4), that is expressed in *ex vivo* cultures of hBM-MSCs [17, 35]. Surface markers, like neuron-glial antigen 2 (NG2), are another category that is upregulated in hBM-MSCs upon cultivation [47]. While CD146 is a pericyte marker [40], SSEA-4 is an embryonic stem cell marker [48] that is used to isolate induced pluripotent stem cells [49]. NG2 potentiates the activity of *β*1 integrins and growth factor receptors in regulating cell proliferation, motility, and survival [50]. hBM-MSCs with high levels of SSEA-4 surface expression proliferate at a faster rate than MSCs with low expression levels for this marker [17]. We observed that the surface expression of CD146 and NG2 is inversely correlated to doubling time during the serial passage of single-cell-derived hBM-MSC cultures [35]. In addition, we observed that the fraction of MSCs with high expression of NG2 and low scatter properties is more clonogenic than the parental MSC culture from which it was derived [35].

### 2.2. Global Molecular Signatures of Proliferation Potential

The global scope of transcriptomic and proteomic profiling can detect differences in cell populations not evident with immunophenotyping [51, 52]. A global molecular signature of MSC heterogeneity may enable greater control over cell composition in MSC therapies than can be achieved with an immunophenotype alone. Microarray analysis of differential gene expression in high- and low-growth populations of hBM-MSCs and human dental tissue-derived (hDT) MSCs identified a common gene signature associated with immature MSCs [19] (Table 2). High-growth MSCs derived from these different tissues exhibited increased expression of genes with critical roles in cell growth and survival: *E2F2*, *PTTG1*, and *TWIST1* are representative of this common signature [19]. The E2F family of transcription factors regulates the G1/S transition of the cell cycle and DNA synthesis, as well as the DNA damage and repair checkpoint response [53]. *PTTG1* (aka pituitary tumor-transforming gene 1) encodes securin, which participates in synchrony of chromosome separation in the anaphase of mitosis [54]. The transcription factor TWIST1 mediates lineage commitment of MSCs and may mediate their self-renewal [55]. hBM-MSCs that were stably transduced with the *TWIST1* gene maintained an immature phenotype and exhibited an increased proliferation rate relative to a mock control [55].

In another study of fast- and slow-growing subsets of hBM-MSCs from osteoarthritis patients, genes encoding sex-determining region Y-box 2 (*SOX2*), notch homolog 1 (*NOTCH1*), and the notch ligand delta-like 3 (*DLL3*) were among the upregulated genes in fast-growing MSCs [56]. These genes are associated with embryonic and postembryonic stem cell renewal [57, 58]. Their expression in fast-growing MSCs is suggestive of an immature phenotype with developmental plasticity [56]. The gene for heat shock 70 kDa protein 9 (aka mortalin or *HSPA9*) was the most overexpressed, by nearly 10-fold, among the genes upregulated in the slow-growing MSC subset. Overexpression of mortalin in senescent normal human lung fibroblasts increased cumulative population doublings, induced a younger cell morphology, and lowered senescence-associated *β*-galactosidase activity relative to controls [59]. Perhaps mortalin upregulation by slow-growing MSCs is an attempt to extend their lifespan.

Proteomic profiling of hBM-MSCs from osteoarthritis patients revealed an overexpression of calcium-binding and actin-binding proteins involved in cytokinesis, such as calmodulin 1 (CALM1) and tropomyosin (TPM4), in fast-growing MSC populations relative to their slow-growing counterparts [60]. Overexpressed proteins in the slow-growing MSCs included heat shock protein 27 (HSP27) and annexin A1 (ANXA1) [60], indicative of cellular stress [61]. Slow-growing MSCs overexpressed caldesmon 1 (CALD1) as well [60]. Caldesmon 1 binds to calmodulin 1 and tropomyosin to inhibit cell division by regulating actin activation of myosin ATPase [62]. This suggests a scenario whereby caldesmon 1 and its targets, calmodulin 1 and tropomyosin, act in a coordinated manner to regulate cell division in MSCs.

## 3. Differentiation Potential

Trilineage potential to exhibit adipo-, chondro-, and osteogenesis is one of the minimal criteria for hMSCs established by the International Society for Cellular Therapy [34]. This criterion pertains to the MSC culture as a whole, which is an ensemble of individual cells with different differentiation potentials. Cellular heterogeneity in the differentiation potential of MSC cultures arises, in part, from progenitors at different stages of lineage commitment, ranging from multi- to unipotent [10, 14, 15]. Initially, lineage commitment was attributed to a sequential loss in differentiation potential [14, 63]. We expanded on this work by developing an *in vitro* high-capacity assay to quantify the clonal heterogeneity in the trilineage potential of hBM-MSCs [15]. By analyzing large numbers of single-cell-derived colonies, we revealed a more complex hierarchy of lineage commitment that results in heterogeneous MSC cultures containing cells with all possible combinations of adipo-, chondro-, and osteogenic potential. RNA sequencing of single cells and single-cell-derived colonies of murine bone marrow-derived (mBM) MSCs validated the inherent complexity of lineage commitment and revealed multiple lineage-specific transcriptional profiles in individual cells and colonies [20, 64].

### 3.1. Cell Surface Markers of Differentiation Potential

Surface markers of proliferation potential can isolate cells that are both rapidly dividing and multipotent from heterogeneous MSC cultures [17, 35, 43]; however, there are limitations to the use of a proliferation biomarker to predict the differentiation potential of MSCs. For instance, a cell subset of hBM-MSCs with an enhanced capacity for chondrogenic differentiation had a similar expression profile for the standard MSC markers (CD73, CD90, and CD105) and proliferation markers (STRO-1, CD146, and CD271) as compared with a cell subset that exhibited low chondrogenic differentiation from the same MSC culture [65]. Likewise, we observed that NG2 surface expression was correlated to the proliferation potential for single-cell-derived hBM-MSC colonies; however, the correlation did not extend to the differentiation potential because tri- and bipotent MSC colonies had similar proliferation potentials [35, 66]. The limitation of a proliferation biomarker to detect some changes in lineage commitment necessitates identifying surface markers whose expression in undifferentiated MSCs is predictive of their differentiation potential.

Much of the research in this area has focused on surface markers of osteogenic and chondrogenic potential given that bone repair and cartilage repair account for nearly 20% of MSC-based clinical trials [13]. Positive activity for tissue nonspecific alkaline phosphatase (TNAP, aka mesenchymal stem cell antigen-1) identified single-cell-derived colonies and sorted groups of undifferentiated hBM-MSCs that had a higher degree of calcium mineralization and greater levels of osteogenic-related genes during *in vitro* osteogenesis than TNAP^−^ MSCs from the same culture [67]. TNAP plays a critical role in the maintenance of bone mineralization by regulating phosphate levels [68, 69]. Expression of TNAP is tissue specific: undifferentiated MSCs from human cord blood (hCB-MSCs) are negative for this marker [67]. As another example, CD271^+^ hBM-MSCs can be divided into a CD56^+^ fraction that is enriched in chondroprogenitors relative to the CD56^−^ fraction [70]. CD56 is a cell adhesion molecule that is coexpressed with CD271 on the bone surface that lines the marrow cavity [70]. Like CD271, CD56 is rapidly downregulated in cultured MSCs [71].

Recently, the receptor tyrosine kinase-like orphan receptor 2 (ROR2) was identified as a predictive marker of chondrogenic potential for hBM-MSCs [65]. Positive selection for ROR2 in confluent cultures of undifferentiated hBM-MSCs isolates a cell subset with an enhanced capacity for chondrogenic differentiation *in vitro* and *in vivo* [65]. Possibly, ROR2 mediates Wnt5a regulation of chondrogenesis by differential use of the nuclear factor of activated T cells (NFAT) and nuclear factor-*κ*B (NF-*κ*B) pathways [72, 73]. ROR2^+^ cells were more prevalent in expanded hBM-MSC cultures from osteoarthritis patients than in control cultures from healthy donors [65]. Consistent with this observation, MSC cultures from the patients produced more cartilage tissue *in vitro* relative to the controls [65].

There is a growing body of evidence that MSCs contain a rare population of pluripotent cells, which have been named multilineage-differentiating stress-enduring (MUSE) cells [74]. MUSE cells have been isolated from mesenchymal tissues by their positive expression of stage-specific embryonic antigen-3 (SSEA-3), a pluripotency marker, and endoglin (CD105), an MSC marker [74]. SSEA-3^+^ CD105^+^ cells account for as little as 1% of hBM-MSCs and 3-9% of hAD-MSCs [74, 75]. MUSE cells express pluripotency factors, such as octamer-binding transcription factor 3/4 (OCT3/4) and SOX2 [74, 76], and exhibit triploblastic differentiation at the single-cell level [77]. This pluripotent phenotype is characteristic of MUSE cells isolated from various tissues, including human bone marrow, skin, and adipose [74, 77, 78]. As their name suggests, MUSE cells are capable of surviving extreme cellular stress [78, 79], which has been exploited to enrich adipose-derived MUSE cells [78]. In addition, MUSE cells from different tissues are nontumorigenic in vivo [74, 75]. Given concerns about induced pluripotent stem cells forming teratomas [80], MUSE cells may potentially provide a safer source of pluripotent cells for clinical applications.

### 3.2. Global Molecular Signatures of Differentiation Potential

RNA sequencing elucidated transcriptional signatures that distinguish between single cell-derived colonies of mBM-MSCs with osteogenic and adipogenic potential [20]. The transcriptional signature for undifferentiated colonies with osteogenic potential included genes for osterix (*Sp7*) and its mediator, distal-less homeobox 5 (*Dlx5*), that are master transcription factors in the bone morphogenetic protein pathway [81]. In contrast, the signature for adipogenic potential contained genes encoding the transcription factors peroxisome proliferator-activated receptor *γ* (*Pparg*) and CCAAT/enhancer-binding protein *α* (*Cebpa*) that regulate each other in a positive feedback loop [20, 82]. Undifferentiated mBM-MSC colonies with both osteogenic and adipogenic potential shared these two distinct gene signatures [20]. Coexistence of distinct transcriptional profiles in MSCs resembles the lineage priming mechanism during early commitment of hematopoietic progenitors [83].

In another study, microarray analysis revealed a transcriptional signature of hBM-MSCs that forms an ectopic ossicle containing both bone and marrow when implanted in immunodeficient mice [84]. Single colony-derived strains of hBM-MSCs are highly variable in their capacity to form this bone/marrow organ [84]. Genes for secreted frizzled-related protein 2 (*SFRP2*) and calponin 1 (*CNN1*) were the most upregulated and downregulated, respectively, in MSC strains that formed a bone/marrow organ relative to those that formed only fibrous tissue [84]. *SFRP2* encodes a soluble inhibitor of Wnt signaling [85], but its involvement in the formation of a bone/marrow organ could extend to other signaling pathways. For example, SFRP2 binds to the fibronectin- (FN-) integrin-*α*5*β*1 complex to inhibit apoptosis [86], which may promote MSC survival in ectopic implants. Calponin 1 is a matrix-binding protein that induces actin polymerization [87]. In regulating cytoskeletal structure, calponin 1 may affect cell proliferation and/or differentiation in MSC implants. Consistent with the downregulation of the *CNN1* gene in bone/marrow-forming MSC strains, *CNN1*-null mice exhibit increased bone formation [88].

## 4. Immunomodulatory and Trophic Properties

MSCs possess extraordinary immunomodulatory and trophic properties to orchestrate endogenous tissue repair. To resolve inflammation in damaged tissue, MSCs suppress inflammation and may promote clearance of inflammatory stimuli [89]. To promote healing, MSCs produce trophic factors to increase angiogenesis and stimulate endogenous cell growth and differentiation [90]. The majority of MSC-based clinical trials utilize immunomodulatory and trophic properties of MSCs to repair damaged tissue of nonmesenchymal origin and tissue injured by inflammation [13]. The first commercial stem cell drug was an MSC therapy—Osiris Therapeutics' Prochymal brand of Remestemcel-L—which is used to treat acute inflammation in graft-versus-host disease [91]. Potential therapeutic benefits of immunomodulation and trophic effects extend to MSC repair of myocardial tissue and other mesenchymal tissues [92, 93]. Given the breadth of these applications, it is critically important to identify molecular profiles of cell-to-cell variation in the immunomodulatory and trophic properties of heterogeneous MSC cultures.

### 4.1. Cell Surface Markers of Immunomodulatory and Trophic Properties

Surface markers that select for colony-forming MSCs, such as CD271 and STRO-1, have been used to enrich MSCs with increased immunosuppressive capacity [94, 95]. In addition to these biomarkers, other antigens have been identified that detect cell-to-cell variation in the immunoregulatory activity of MSCs [96–98]. Of these, the expression of vascular cell adhesion molecule-1 (VCAM-1, CD106) in MSCs has been extensively studied [98–100]. Cell-cell adhesion via VCAM-1 plays a key role in the MSC regulation of T cells: MSC-induced suppression of T-cell proliferation correlates with T-cell adhesion to MSCs and is reversed with blocking VCAM-1 antibody [100]. When MSCs derived from human term placental chorionic villi (hCV-MSCs) were divided into VCAM-1^−^ and VCAM-1^+^ subsets, the latter was far more effective in (1) suppressing the secretion of inflammatory cytokines by activated peripheral blood mononuclear cells and (2) stimulating the formation of anti-inflammatory regulatory T cells [98]. The content of VCAM-1^+^ cells in MSC cultures is tissue specific: more in hCV-MSCs than in hBM-MSCs and none in hAD-MSCs [98]. The high levels of immunosuppressive VCAM-1^+^ cells in hCV-MSCs may be associated with fetomaternal tolerance. The placenta provides an immune-privileged environment for the fetus [101]. VCAM-1^+^ MSCs within chorionic villi may help suppress an immune response at the border between maternal and fetal blood.

A growing body of data suggests that MSCs may exhibit proinflammatory properties in certain instances, perhaps to stimulate pathogen clearance [89, 102]. Recently, the surface marker tetherin (aka bone marrow stromal antigen 2 and CD317) identified a subset (~1%-3%) of hBM-MSCs with elevated secretion of interleukin-7 (IL-7) [96], which mediates B and T cell development [103]. As its name suggests, tetherin inhibits the spread of viral infection by tethering budding viruses to infected cells [104]. In addition, tetherin activates NF-*κ*B to induce an inflammatory response [105]. Tetherin^+^ MSCs could potentially have a role in the clearance of viral infections. It is noteworthy that the differentiation potential of tetherin^+^ hBM-MSCs was compromised relative to tetherin^−^ MSCs from the same culture [96], suggesting that there are limitations to using differentiation markers to select for MSCs with specific immunomodulatory properties.

A few surface markers have been examined for their ability to isolate MSCs with enhanced trophic activity to increase angiogenesis and promote endogenous tissue repair. For instance, murine gastrocnemius muscle injected with CD146^+^ hCV-MSCs had more blood vessels at the injection site than controls receiving the CD146^−^ cell fraction [106]. Cell-to-cell variation in the trophic activity of STRO-1^+^ hBM-MSCs was evident when they were divided into STRO-1^Bright^ and STRO-1^Dim^ subsets: conditioned medium from the STRO-1^Bright^ MSCs elicited greater cardiac cell proliferation and endothelial tube formation [107]. Inhibition of stromal cell-derived factor 1 (SDF1, CXCL12) and hepatocyte growth factor (HGF) in the conditioned medium attenuated these trophic effects [107].

### 4.2. Global Molecular Signatures of Immunomodulatory and Trophic Properties

Microarray and qPCR analysis of VCAM-1^+^ hCV-MSCs provided insight into the enhanced anti-inflammatory potential of this cell subset [98]. Relative to VCAM-1^−^ hCV-MSCs, the VCAM-1^+^ subset had elevated mRNA levels of cyclooxygenase-2 (*COX2*), indoleamine 2,3-dioxygenase 1 (*IDO1*), and other key immune modulators, as well as secreted more prostaglandin E2 (PGE2) protein [98]. While COX-2 is typically associated with inflammation [108], its increased expression in VCAM-1^+^ MSCs may have an anti-inflammatory function. Specifically, COX-2 may increase the production of the immunosuppressors PGE2 [109] and IDO-1 [110] in MSCs. In support of this possibility, COX-2 is the rate-limiting enzyme in PGE2 synthesis [111] and regulates IDO-1 expression in animal models of cancer [112]. Overexpression of COX-2 increases the immunosuppressive activity of MSCs derived from human umbilical cord [113].

Interrogation of the transcriptome of tetherin^+^ hBM-MSCs revealed a gene signature that is consistent with a possible role for this cell subset in viral clearance [96]. Single colony-derived strains of tetherin^+^ hBM-MSCs expressed several antiviral genes, including interferon-stimulated gene 20 kDa protein (*ISG20*) and oligoadenylate synthetase (*OAS*), at higher levels than the parental culture and tetherin^−^ strains of hBM-MSCs [96]. Both gene products are induced by interferon and involved in the destruction of RNA viruses: ISG20 is a secreted RNase that directly attacks the viruses [114], and OAS acts indirectly by activating intercellular RNase L [115]. The antiviral activity of ISG20 and OAS complements the ability of tetherin to sequester virus particles [104] and induce an inflammatory response [105].

Proteomic analysis of conditioned medium from hBM-MSCs detected uniquely secreted trophic factors for angiogenesis in cell subsets with elevated expression of aldehyde dehydrogenase (ALDH) [116]. A fluorescent substrate enables cell enrichment based on intercellular ALDH activity [117]. Conditioned medium from ALDH^Bright^ MSCs stimulated *in vitro* endothelial cell proliferation and tube formation and *in vivo* angiogenesis to a greater extent than ALDH^Dim^ MSCs from the same culture [116]. While the ALDH^Bright^ and ALDH^Dim^ subsets had similar gene signatures, their secretome was different [116]. ALDH^Bright^ MSCs uniquely secreted several proangiogenic cytokines, which included vascular endothelial growth factor *β* (VEGFB) and platelet derived growth factor *α* (PDGFA); whereas, ALDH^Dim^ MSCs secreted inhibitors of angiogenesis, such as platelet factor 4 (PF4) and plasminogen (PLG) [116]. The coexistence of pro- and antiangiogenic MSC subsets in the same culture could be akin to an angiogenic on/off switch evident during tumorigenesis [118]. MSC subsets that induce angiogenesis could promote vessel sprouting; whereas, subsets that are inhibitory could cause vessel trimming during vascular remodeling [119].

## 5. Cellular Aging

Cellular aging and replicative senescence compromises stem cell fitness and is an obstacle to the production of effective MSC therapies. Replicative stress caused by rapid MSC expansion during biomanufacturing can induce DNA damage and cellular aging [120]. During serial passage, MSCs experience a continuous and organized aging process [121] that diminishes their regenerative potential and culminates in replicative senescence [122, 123]. Cellular aging also can occur *in vivo* prior to harvesting MSCs from a donor. Repeated cell division during tissue maintenance and repair can cause cellular aging over the life of the donor, particularly in the elderly [120]. The negative impact of cellular aging on MSC therapies is twofold. First, the loss of stem cell fitness limits, if not prevents, *ex vivo* expansion to produce clinically relevant quantities of MSCs. Second, it may impair the effectiveness of MSC therapies to regenerate damaged tissue. We and others have observed that MSC cultures contain a heterogeneous mixture of cells at different stages of aging, starting at early passage [16, 17, 124]. Molecular profiles of this heterogeneity would be useful to develop expansion conditions that mitigate cellular aging, as well as to monitor and control the cell composition of MSC therapies during biomanufacturing.

### 5.1. Cell Surface Markers of Cellular Aging

Surface markers for rapidly dividing, multipotent MSCs (e.g., CD146, NG2, and SSEA-4) are downregulated in aging MSCs during *ex vivo* expansion [17, 35]. For example, Rosu-Myler et al. [17] described early passage hBM-MSCs as a heterogeneous mixture of cells with high and low surface expression of SSEA-4. Serial passage on tissue culture plastic depletes clonal, multipotent SEEA-4^hi^ MSCs from culture and causes an accumulation of SSEA-4^lo^ cells with diminished regenerative potential [17]. Block et al. [124] reported that small, SSEA-4^+^ cells accounted for less than 10% of hBM-MSCs harvested from elderly donors ages 65 and older. They were able to isolate these high-quality cells and expand them on the extracellular matrix from young-donor MSCs to quantities required for clinical applications [124].

A surface marker expressed on aging cells would enable the enrichment of rapidly dividing MSCs by negative selection. Recently, we reported on decoy TRAIL receptor CD264 as the first surface marker of cellular aging for MSCs [16]. The content of CD264^+^ cells accumulates during serial passaging of hBM-MSC cultures: CD264 is initially upregulated at an intermediate passage concurrently with p21 and remains upregulated at late passage as aging progresses to senescence [16]. There is a strong inverse correlation of CD264^+^ cell content with multiple metrics of stem cell fitness, including colony-forming efficiency as a measure of proliferation potential [16]. MSCs may increase CD264 expression to promote cell survival during aging possibly via the protein kinase B (aka Akt) signaling pathway [125].

### 5.2. Global Molecular Signatures of Cellular Aging

Gene and epigenetic signatures have been identified for molecular changes in heterogeneous MSCs associated with replicative senescence [126, 127], but to date there are no senescence signatures for cell subsets in MSC cultures. It should be noted that senescence signatures developed for heterogeneous MSCs represent an ensemble average of molecular changes for individual cells within the bulk culture. As such, they may not be predictive of the properties of specific cell subsets. To emphasize this point, the epigenetic senescence signatures for heterogeneous hBM-MSC cultures and their subclones were compared recently [128]. The signature consists of DNA methylation changes at six CpG sites, which predicts the average passage number of heterogeneous hBM- and hAD-MSC cultures [127]. While the DNA methylation pattern provided a good correlation between predicted and real passage number of the bulk hBM-MSC cultures, the predictions did not correlate at all with the real passage number of the subclones [128]. Moreover, the differentiation potential of the subclones did not correlate with their senescence signature [128]. This example illustrates the need for global senescence signatures that are relevant to cell subsets, not just the bulk culture.

## 6. Applications

**Figure 1:**
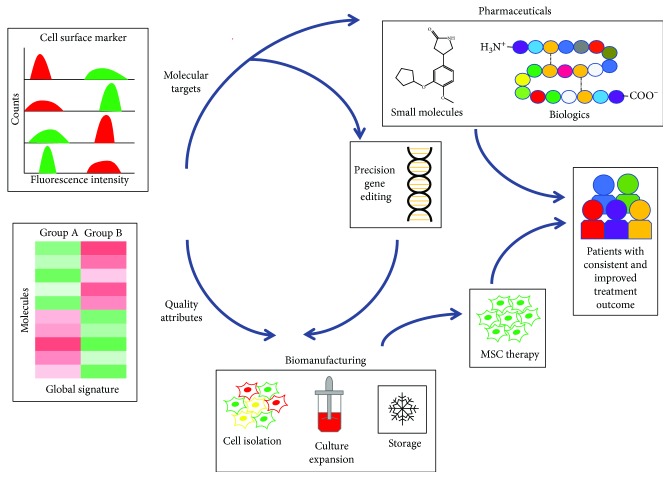
Applications of molecular profiles of MSC heterogeneity. Surface markers and global signatures identify cell subsets with specific regenerative properties in heterogeneous MSC cultures. Molecular profiles of MSC heterogeneity have application as molecular targets and quality attributes in the production of MSC therapeutics. Targeted molecules can be regulated by small-molecule and biologic pharmaceuticals, as well as by precision gene editing. Quality attributes enable enrichment of a MSC population and its assessment during all stages of biomanufacturing of MSC therapies from cell isolation to culture expansion to storage. This control over the composition and function of MSC therapies has the potential to improve treatment outcomes for patients.

The molecular profiles described here provide new insight into MSC biology. This knowledge can be exploited to identify molecular targets to regulate the regenerative potential of MSCs (Figure 1). Targeted molecules and pathways can be regulated by chemical and biologic agents administered as adjuvants with MSC therapies. For example, we demonstrated that a small-molecule antagonist of macrophage migration inhibitory factor enhances the migratory response of hBM-MSCs to injured bronchial epithelial cells [129]. Matrix proteins when administered as an adjuvant with hBM-MSCs increase *in vivo* survival of the stem cells by attenuating anoikis [130]. Recent improvements in the nonviral delivery of CRISPR/Cas9 components have enabled safe and effective gene editing of hard-to-transfect MSCs [131]. Adjuvant therapies and precision gene editing inspired from MSC molecular profiles have the potential to improve the effectiveness of MSC therapies by providing unprecedented control over their regenerative potential.

Surface markers and global signatures of MSC heterogeneity have the potential to be effective in predicting treatment outcome in MSC-based clinical trials. Predictive molecular profiles of the regenerative potential of MSCs have utility as quantifiable attributes of cell quality during the manufacturing of MSC therapies [132]. These quality attributes can enable enrichment of a MSC population and its assessment during all stages of manufacturing from the selection of the source stem cell to the preparation of the final clinical-grade product. Most likely, MSC quality attributes will be multivariate and specific to the tissue of origin, with a unique combination of surface markers and global signatures for each application. For example, they could include CD271 and other tissue-specific markers of multipotency [39]; negative markers of an undesirable phenotype, such as cellular aging [16]; and global signatures that can classify MSCs based on a specific function, like immunomodulation [116]. When measured in real time, these quality attributes can enable feedforward and feedback control of the manufacturing process [133] to improve the consistency and quality of an MSC therapy.

## 7. Future Directions

Molecular profiles of MSC heterogeneity are far from complete. More research is needed in all categories of MSC function and, in particular, homing. Upon remote delivery of MSC therapies to a patient, the stem cells are capable of homing to the site of injury. Despite its importance to tissue repair, little is known about the cell-to-cell variation in the ability of MSCs to home to injured tissue [134]. To date, most of the global profiles of MSC heterogeneity have been gene signatures, with limited analysis of the proteome and secretome. In the future, more comprehensive profiling is warranted that includes miRNA, lncRNA, and the epigenome, given their roles in the regulation of stem cell function [135–137].

There is limited information on how the molecular profiles of MSC heterogeneity are influenced by variables encountered during the manufacturing of MSC therapies. MSC heterogeneity can be profoundly affected by the choice of stem cell donor [65], tissue source [98], and process conditions [16]. Donor variables include age, BMI, sex, and health status. As reflected in this review, popular tissue sources for MSCs are bone marrow and adipose, and there is a growing interest in perinatal tissues as a source of primitive MSCs that can be harvested noninvasively. Process conditions, such as passage number and confluency, determine the extent of cell expansion during the manufacturing of MSC therapies. An understanding of the influence of production variables on the surface markers and global signatures described in this review is essential for these molecular profiles to be adopted as quality attributes during the manufacturing of MSC therapies. This knowledge can guide the selection of donor and tissue source and the design of process conditions that optimize MSC performance in the clinic.

Many of the molecular profiles discussed here have yet to be validated in an animal model. *In vitro* behavior of MSCs is not a reliable predictor of their *in vivo* performance. As a case in point, only a fraction of clonal mBM-MSCs, which exhibit *in vitro* osteogenesis, are capable of *in vivo* bone formation [20]. Promising surface markers and global signatures that have been identified based on *in vitro* function of MSCs need to be validated first in animal models and then in clinical trials. Molecular profiles that are predictive of clinical outcome are candidates to use as quality attributes for robust and reproducible manufacturing of MSC therapies.

## 8. Conclusions

Inconsistencies in the composition of MSC cultures hinder their use in regenerative medicine. There is a critical need for molecular profiles of this heterogeneity to manufacture effective MSC therapies. This review presented cell surface markers and global signatures that isolate cell subsets with specific regenerative properties from heterogeneous MSC cultures. More research is required to ascertain how these molecular profiles are influenced by biomanufacturing conditions. Other areas to be explored include noncoding RNA and epigenetic signatures, in addition to the in vivo validation of many of the molecular profiles. Predictive surface markers and global signatures of regenerative potential will facilitate robust and reproducible manufacturing of MSC therapies, as well as identify new molecular targets to regulate MSC function. This control over MSC composition and function will accelerate the translation of MSC therapeutics into clinical practice.

## Figures and Tables

**Table 1:** Representative surface markers of cell subsets for heterogeneous MSCs.

Cell subset	Surface marker	Comments	References
Fast growing/multipotent MSCs	Low-affinity nerve growth factor receptor (CD271)	Isolation marker that is downregulated in expanded MSCs	[39, 43, 44, 46, 94]
	Melanoma cell adhesion molecule (CD146)	A pericyte marker expressed in primary and expanded MSCs	[35, 38, 40, 95, 106]
	Neuron-glial antigen 2 (NG2)	Upregulated in expanded MSCs. Similar expression in tri- and bipotent MSCs	[35, 47]
	Stage-specific embryonic antigen-4 (SSEA-4)	An embryonic stem cell marker expressed on primitive MSCs	[17, 48, 49]
	STRO-1	Clonogenic MSCs constitute a small fraction of the isolated stromal cells	[36, 37, 107]

Osteogenic MSCs	Tissue nonspecific alkaline phosphatase (TNAP)	Selects for MSCs with increased mineralization and expression of osteogenic-related genes	[67]

Chondrogenic MSCs	Neural cell adhesion molecule (CD56)	Isolates chrondroprogenitors but is downregulated in expanded MSCs	[70, 71]
	Receptor tyrosine kinase-like orphan receptor 2 (ROR2)	Isolates chrondroprogenitors from confluent, undifferentiated MSCs	[65]

Triploblastic MUSE cells	Stage-specific embryonic antigen-3 (SSEA-3)	Selected cells exhibit triploblastic differentiation at the single-cell level	[74–79]

Immunoregulatory MSCs	Tetherin (bone marrow stromal antigen 2, CD317)	MSCs isolated for tetherin expression have proinflammatory properties and may participate in pathogen clearance	[96]
	Vascular cell adhesion molecule-1 (CD106)	Selects MSCs that suppress inflammatory cytokine and stimulate regulatory T cells	[98–100]

Aging MSCs	Decoy TRAIL receptor (CD264)	Upregulated concurrently with p21 and remains elevated through senescence	[16]

MSCs: mesenchymal stem cells. MUSE: multilineage-differentiating stress enduring.

**Table 2:** Global molecular signatures of cell subsets in heterogeneous MSC cultures.

Cell subset	Molecular signature	MSC source	Reference
Fast growing/multipotent relative to slow-growing MSCs	Upregulated genes: *ASPM*, *AURKB*, *CCNB2*, *CDC2*, *CDC20*, *CENPF*, *CEP55*, *CHEK1*, *CIT*, *CKS2*, *DLG7*, *E2F2*, *GINS2*, *LDB2*, *MAD2L1*, *NCAPG*, *PBK*, *POLQ*, *PTTG1*, *RPA3*, *RRM2*, *TOP2A*, *TWIST1*, *UBE2C*	Human bone marrow and dental tissue	[19]

Fast growing/multipotent MSCs	Upregulated genes: *ACAN*, *ALP1*, *BMP2*, *CDC2*, *CDH1*, *COL1A1*, *COL2A1*, *DLL3*, *DVL1*, *FGF2*, *FOXA2*, *GDF2*, *IGF1*, *JAG1*, *NEUROG2*, *NOTCH1*, *SOX2*	Human bone marrow	[56]

Slow-growing MSCs	Upregulated genes: *ALDH1A1*, *CCND2*, *CD44*, *DTX1*, *FGF1*, *HSPA9*, *MSX1*, *TUBB3*		

Fast growing/multipotent MSCs	Upregulated proteins: CALM1, POMC, TPM4	Human bone marrow	[60]

Slow-growing MSCs	Upregulated proteins: ANXA1, CALD1, ENO1, GAPDH, HSP27, LMNA, PKM		

Osteogenic MSCs	Upregulated genes: *Col1a1*, *Col1a2*, *Comp*, *Dlx3*, *Dlx5*, *Fgfr3*, *Fmod*, *Gli1*, *Hey1*, *Ibsp*, *Pitx1*, *Prrx2*, *Ptch1*, *Pth1r*, *Ror2*, *Sp7*, *Tbx3*	Mouse bone marrow	[20]

Adipogenic MSCs	Upregulated genes: *Abca1*, *Abcg1*, *Cebpa*, *Ctgf*, *Cxcl12*, *Dlk1*, *Foxc2*, *Inhbb*, *Lpl*, *Nr1h3*, *Pgf*, *Plin4*, *Pparg*, *Prdm16*, *Sox5*, *Ucp2*		

Marrow-forming MSCs relative to MSCs that form fibrous tissue	Upregulated genes: *ASPN*, *BMP2*, *BMP4*, *CXCL1*, *DCN*, *EYA1*, *GNAS*, *ICAM1*, *IGF1*, *IL8*, *MEOX2*, *MN1*, *MSX2*, *OGN*, *OMD*, *PRRX1*, *SFRP2*, *WISP1*	Human bone marrow	[84]
	Downregulated gene: *CNN1*		

Immunomodulatory, VCAM-1^+^ relative to VCAM-1^−^ MSCs	Upregulated genes: *COX2*, *IDO1*, *IL1A*, *IL1B*, *IL6*, *IL8*	Human term placental chorionic villi	[98]
	Upregulated secreted protein: PGE2		

Immunomodulatory, tetherin^+^ relative to tetherin^−^ MSCs	Upregulated genes: *ADAR*, *BST2*, *EIF2AK2*, *IL18*, *ISG15*, *ISG20*, *MX1*, *MX2*, *OAS1*, *OAS2*, *OAS3*, *OASL*	Human bone marrow	[96]

Trophic activity, ALDH^Bright^ MSCs	Uniquely secreted proteins: ACVR1, ANG, GREM1, IGF1, METRN, PDGFA, PLXND1, SPON1, VEGFB, WNT5A	Human bone marrow	[116]

Trophic activity, ALDH^Dim^ MSCs	Uniquely secreted proteins: ANGPTL3, APOH, BMP2, MMP19, PF4, PLG, PTPRM, PTPRU, TIE1		

MSCs: mesenchymal stem cells. Nomenclature for global molecular signatures is provided in the supplementary material ([Supplementary-material supplementary-material-1]).

## Supplementary Materials

Supplementary Materials Supplementary Table: nomenclature for global molecular signatures.
