# Supplementary material for: Molecular Profiles of Cell-to-Cell Variation in the Regenerative Potential of Mesenchymal Stromal Cells
Source: Stem Cells Int. 2019 Sep 17;2019:5924878. doi: 10.1155/2019/5924878 (PMC6766122; doi:10.1155/2019/5924878)
Supplement: Supplementary Materials — Supplementary Table: nomenclature for global molecular signatures. [file 5924878.f1.pdf]

SUPPLEMENTARY TABLE: Nomenclature for global molecular signatures.

Symbol	Protein name	Symbol	Protein name
ABCA1	ATP binding cassette subfamily A member 1	COX2	Cytochrome c oxidase subunit II, cyclooxygenase 2
ABCG1	ATP binding cassette subfamily G member 1	CTGF	Connective tissue growth factor
ACAN	Aggrecan	CXCL1	C-X-C motif chemokine ligand 1
ACVR1	Activin A receptor type 1	CXCL12	C-X-C motif chemokine ligand 12
ADAR	Adenosine deaminase RNA specific	DCN	Decorin
ALDH1A1	Aldehyde dehydrogenase 1 family member A1	DLG7	Discs large homolog 7
ALP1	Alkaline phosphatase 1	DLK1	Delta like non-canonical Notch ligand 1
ANG	Angiogenin	DLL3	Delta-like 3
ANGPTL3	Angiopoietin like 3	DLX3	Distal-less homeobox 3
ANXA1	Annexin A1	DLX5	Distal-less homeobox 5
APOH	Apolipoprotein H	DTX1	Deltex homolog 1
ASPM	Asp (abnormal spindle) homolog	DVL1	Dishevelled, dsh homolog 1
ASPN	Asporin	E2F2	E2F transcription factor 2
AURKB	Aurora kinase B	EIF2AK2	Eukaryotic translation initiation factor 2 alpha kinase 2
BMP2	Bone morphogenetic protein 2	ENO1	Enolase 1
BMP4	Bone morphogenetic protein 4	EYA1	Eyes absent homolog 1
BST2	Bone marrow stromal cell antigen 2	FGF1	Fibroblast growth factor 1 (acidic)
CALD1	Caldesmon 1	FGF2	Fibroblast growth factor 2 (basic)
CALM1	Calmodulin 1	FGFR3	Fibroblast growth factor receptor 3
CCNB2	Cyclin B2	FMOD	Fibromodulin
CCND2	Cyclin D2	FOXA2	Forkhead box A2
CD44	CD44 molecule	FOXC2	Forkhead box C2
CDC2	Cell division cycle 2	GAPDH	Glyceraldehyde 3-phosphate dehydrogenase
CDC20	Cell division cycle 20 homolog	GDF2	Growth differentiation factor 2
CDH1	Cadherin 1, type 1, E-cadherin	GINS2	GINS complex subunit 2
CEBPA	CCAAT/enhancer binding protein alpha	GLI1	GLI family zinc finger 1
CENPF	Centomere protein F	GNAS	GNAS complex locus
CEP55	Centrosomal protein 55 kDa	GREM1	Gremlin 1, DAN family BMP antagonist
CHEK1	Checkpoint kinase 1	HEY1	Hairy/enhancer-of-split related with YRPW motif 1
CIT	Citron Rho-interacting kinase	HSP27	Heat shock protein 27
CKS2	CDC28 protein kinase regulatory subunit 2	HSPA9	Heat shock 70 kDa protein 9, mortalin
CNN1	Calponin 1	IBSP	Integrin binding sialoprotein
COL1A1	Collagen type I alpha 1	ICAM1	Intercellular adhesion molecule 1
COL2A1	Collagen type II alpha 1	IDO1	Indoleamine 2,3-dioxygenase 1
COMP	Cartilage oligomeric matrix protein	IGF1	Insulin-like growth factor 1

(continued)

SUPPLEMENTARY TABLE (CONTINUED)

Symbol	Protein name	Symbol	Protein name
IL1A	Interleukin 1 alpha	PITX1	Paired like homeodomain 1
IL1B	Interleukin 1 beta	PKM	Pyruvate kinase isozymes M1/M2
IL6	Interleukin 6	PLG	Plasminogen
IL8	Interleukin 8	PLIN4	Perilipin 4
IL18	Interleukin 18	PLXND1	Plexin D1
INHBB	Inhibin subunit beta B	POLQ	DNA polymerase theta
ISG15	Interferon-stimulated gene 15 kDa protein	POMC	Pro-opiomelanocortin
ISG20	Interferon-stimulated gene 20 kDa protein	PPARG	Peroxisome proliferator-activated receptor gamma
JAG1	Jagged 1	PRDM16	PR/SET domain 16
LDB2	LIM domain-binding 2	PRRX1	Paired related homeobox 1
LMNA	Lamin A/C	PRRX2	Paired related homeobox 2
LPL	Lipoprotein lipase	PTCH1	Patched 1
MAD2L1	MAD2 mitotic arrest deficient 2 like 1	PTH1R	Parathyroid hormone 1 receptor
MEOX2	Mesenchyme homeobox 2	PTPRM	Protein tyrosine phosphatase receptor type M
METRN	Meteorin, glial cell differentiation regulator	PTPRU	Protein tyrosine phosphatase receptor type U
MMP19	Matrix metallopeptidase 19	PTTG1	Securin, pituitary tumor-transforming 1
MN1	Meningioma 1	ROR2	Receptor tyrosine kinase like orphan receptor 2
MSX1	Msh homeobox 1	RPA3	Replication protein A3
MSX2	Msh homeobox 2	RRM2	Ribonucleotide reductase M2
MX1	MX dynamin like GTPase 1	SFRP2	Secreted frizzled-related protein 2
MX2	MX dynamin like GTPase 2	SOX2	Sex-determining region Y-box 2
NCAPG	Non-SMC condensin I complex subunit G	SOX5	Sex-determining region Y-box 5
NEUROG2	Neurogenin 2	SP7	Sp7 transcription factor
NOTCH1	Notch homolog 1	SPON1	Spondin 1
NR1H3	Nuclear receptor subfamily 1 group H member 3	TBX3	T-box transcription factor 3
OAS1	2'-5'-Oligoadenylate synthetase 1	TIE1	Tyrosine kinase 1
OAS2	2'-5'-Oligoadenylate synthetase 2	TOP2A	Topoisomerase (DNA) II alpha
OAS3	2'-5'-Oligoadenylate synthetase 3	TPM4	Tropomyosin alpha-4 chain
OASL	2'-5'-Oligoadenylate synthetase like	TUBB3	Tubulin, beta 3
OGN	Osteoglycin	TWIST1	Twist homolog 1
OMD	Osteomodulin	UBE2C	Ubiquitin-conjugated enzyme E2C
PBK	PDZ-binding kinase	UCP2	Uncoupling protein 2
PDGFA	Platelet derived growth factor subunit alpha	VCAM1	Vascular cell adhesion molecule 1
PF4	Platelet factor 4	VEGFB	Vascular endothelial growth factor beta
PGE2	Prostaglandin E2	WISP1	WNT1 inducible signaling pathway protein 1
PGF	Placental growth factor	WNT5A	Wnt family member 5A
